# Mechanisms of Podocyte Injury in Kidney Allografts

**DOI:** 10.34067/KID.0000001091

**Published:** 2025-12-05

**Authors:** Gabriel C. Barsotti, Madhav C. Menon, Abhijit Naik, Arundati Rao

**Affiliations:** 1 Division of Internal Medicine, Section of Nephrology, Yale University School of Medicine, New Haven, Connecticut; 2 Division of Nephrology, Department of Internal Medicine, University of Michigan School of Medicine, Ann Arbor, Michigan

**Keywords:** chronic allograft failure, chronic GN, glomerular hyperfiltration, kidney transplantation, podocyte, proteinuria, transplant outcomes, transplantation

## Abstract

Long-term kidney allograft survival remains an important clinical challenge. Although immune-mediated injury, particularly antibody mediated rejection, is associated with long-term graft loss, nonimmune mechanisms also drive chronic allograft failure. Podocytes are essential components of the glomerular filtration barrier, and their injury in the transplant setting may accelerate graft loss. In this review, we will discuss mechanisms of podocyte injury contributing to chronic graft failure including immune injury and nonimmune mechanisms such as immunosuppressive drug exposure and transplant-specific biomechanical changes.

## Introduction

Long-term kidney allograft loss remains a clinical challenge. Multiple immune and nonimmune mechanisms drive chronic allograft failure, suggesting the need for integrated strategies to improve graft survival. Biopsy studies link donor-specific antibodies (DSA), microvascular injury (MVI), and interstitial fibrosis and are with late graft loss, highlighting the central role of immune-mediated mechanisms.^1^ However, other studies have identified high rates of arteriolar hyalinosis, mesangial sclerosis, and glomerulosclerosis (GS) later post-transplant, such as progressive native kidney disease, emphasizing likely shared nonimmunologic injury pathways^2^ in allograft loss.

### Role of Podocyte Injury/Depletion in Allograft Loss

Podocyte depletion remains as an important mechanism driving progression of chronic allograft injury, despite advances in immunosuppression.^3^ Podocytes are specialized, terminally differentiated neuron-like epithelial cells that cover the outer surface of the glomerular basement membrane (GBM) and form a critical component of the glomerular filtration barrier (reviewed in^4–6^). Situated at the interface of the urinary space, basement membrane, and vascular compartment, podocytes transduce mechanical, metabolic, and immune signals to maintain selective capillary permeability.^6^ Their interdigitating foot processes constitute the slit diaphragm preventing macromolecular leakage into the urinary space.^5^ Far from being passive structural elements, podocytes engage in dynamic crosstalk with neighboring cells. Endothelial interactions mediated by vascular endothelial growth factor and endothelin-1 are essential for barrier stability, but their dysregulation drives GS.^7–9^ Under inflammatory conditions, podocytes may upregulate danger associated molecular signals activating both innate as well as adaptive immune response involving CD8^+^ *T* cells, characteristics that carry greater relevance in an allograft.^10^ Podocytes are particularly vulnerable to mechanical injury.^11,12^ An early structural hallmark of injury is foot process effacement (FPE), a reversible change associated with actin cytoskeleton remodeling.^13^ With persistent damage, FPE progresses to podocyte detachment and depletion, a largely irreversible process.^14^ Podocyte loss compromises the filtration barrier and imposes biomechanical stress on remaining cells, inducing compensatory hypertrophy that, when maladaptive, perpetuates a cycle of injury. Experimental models indicate that a critical depletion threshold of 20%–40% triggers GS and progression to ESKD.^15–22^ In homeostasis, podocytes display high levels of autophagy, which provides energy, removes damaged organelles, and maintains cellular integrity,^23^ which is regulated by AMP-activated protein kinase signaling,^24–28^ and mechanistic target of rapamycin complex (mTORC1).^29–32^ Multiple experimental^33^ and clinical data demonstrate that mTORC1 signaling may be relevant to compensatory hypertrophy that allografts are forced to sustain. Taken together, podocytes are central in maintaining the glomerular filtration barrier but remain susceptible to immune, mechanical, and metabolic stressors. In kidney allografts, these vulnerabilities are amplified by alloimmune responses, immunosuppressive drug exposure, and transplant-specific biomechanical changes, indicating podocyte injury as a common pathway leading to GS and long-term graft loss, a mechanism well validated in native kidneys but not fully elucidated in the transplant setting.

### Mechanisms of Podocyte Injury in Allografts

#### Immune Mechanisms

In the context of allografts, immunologic mechanisms of podocyte injury are likely paramount. Injury to podocytes is clinically characterized by proteinuria and structurally by podocyte FPE, podocyte loss, and/or GS.

#### Autoimmune Podocyte Injury

Autoimmune podocyte injury in kidney allografts reflects recurrence of native or *de novo* glomerular diseases, where pathogenic antibodies or immune dysregulation reproduce the same podocytopathy lesions in the graft, leading to proteinuria and allograft loss. Minimal change disease (MCD), childhood idiopathic nephrotic syndrome (INS), and primary FSGS are immune-mediated podocytopathies that present with nephrotic-range proteinuria. The efficacy of B-cell–targeted therapy in MCD supports an antibody-mediated mechanism. In 2021, Ye *et al*.^34^ reported podocyte autoantibodies in 66% of children with INS; seven antibodies were most closely linked to pathogenesis, antibody levels correlated with proteinuria, and seropositive biopsies most often showed MCD or FSGS. Nephrin autoantibodies are detectable during active MCD and fall with treatment,^35^ and antinephrin antibodies track disease activity in MCD/INS.^36^ Pretransplant antinephrin positivity also predicts post-transplant FSGS recurrence (hazard ratio [HR], 4.9; *P* < 0.001).^37^ In the multicenter Post-Transplant Glomerular Disease (TANGO) study, a cohort of 176 adult kidney transplants for idiopathic FSGS, recurrence occurred in 32%, typically very early (median 1.5 months post-transplant), and conferred an approximately five-fold higher risk of graft loss (adjusted HR 4.80); 39% of recurrent cases lost the graft versus 15% without recurrence.^38^

Membranous nephropathy (MN), most often primary, presents with nephrotic syndrome. In 2009, podocyte antigen phospholipase A2 receptor (PLA2R) was identified as the major target of pathogenic autoantibodies in primary MN.^39^ Anti-PLA2R titers aid prognostication: high or rising titers correlate with persistent nephrotic syndrome, worsening kidney function, and may prompt immunosuppression.^40^ Antibody dynamics help guide therapy, as summarized by Tesar and Hruskova.^41^ Other podocyte antigens include THSD7A,^42^ NELL-1,^43^ semaphorin 3B,^44^ and HTRA1.^45^ After transplantation, incidence of MN recurrence is 31% at 10 years. Recurrence is strongly predicted by pretransplant anti-PLA2R positivity (HR, 18.58), with titers >150 RU/ml linked to earlier recurrence. Notably, medium-term graft outcomes are similar between recurrent and nonrecurrent MN: allograft failure was 9% in both groups (10-year graft survival 87% versus 90%).^46^

Lupus nephritis (LN) remains an important cause of ESKD, and kidney transplantation is the preferred therapy^47^; however, LN can recur post-transplant and affect allograft outcomes. In a multisite cohort of LN kidney transplants, recurrent LN (RLN) was identified in 35.7% of recipients evaluated with histology,^48^ more often in Black than White recipients (48.3% versus 29%). While 5-year allograft survival did not differ between RLN and non-RLN, survival curves diverged after approximately 6 years with worse survival in RLN group. Overall, allograft failure occurred in 30% with RLN versus 25% without.^48^ Another manifestation of LN is lupus podocytopathy, which presents with nephrotic syndrome and typically shows MCD, FSGS, or mesangial proliferation on biopsy with Ig and complement deposition.^21^ Lupus podocytopathy is currently considered predominantly *T*-cell–mediated rather than antibody-driven.^49^

#### Post-Transplant Diabetes Mellitus

Post-transplant diabetes mellitus (PTDM) shares common risk factors and pathologic features with diabetic kidney disease,^50,51^with hemodynamic and metabolic stress driving glomerular changes, such as mesangial expansion, thickening of the GBM, and podocyte injury, leading to GS, proteinuria, and disease progression.^50,52^

PTDM can affect 4%–25% of kidney transplant recipients (KTRs),^53,54^ typically appearing within the first post-transplant year.^55–58^ Its pathophysiology is similar to type 2 diabetes, involving insulin resistance, excessive hepatic glucose production, *β*-cell dysfunction, and disruption of the incretin axis,^59–62^ although renal gluconeogenesis has not been demonstrated.^63^ However, immunosuppression is the major risk factor for the development of PTDM.

In contrast to patients with advanced CKD, who are prone to hypoglycemia due to impaired drug clearance, reduced renal gluconeogenesis, and a weakened counter-regulatory response,^64^ KTRs typically regain near-normal kidney function, combined with diabetogenic properties of immunosuppressants such as glucocorticoids,^65–68^ calcineurin inhibitors (CNIs),^69–72^ and mammalian target of rapamycin inhibitors (MTORis)^73–77^ shifts the metabolic balance toward hyperglycemia promoting PTDM. Evidence on PTDM's impact on outcomes is mixed. In a multicenter study over 6 years, there was no increase in graft failure or mortality^78^; however in a recent study, there was increased risk of all-cause mortality [risk ratio, 1.70; 95% confidence interval, 1.53 to 1.89; *P* < 0.001] and of graft failure (risk ratio, 1.33; 95% confidence interval, 1.16 to 1.54; *P* < 0.001).^79^ In this context, newer agents such as sodium-glucose cotransporter 2 (SGLT2) inhibitors and glucagon-like peptide (GLP) agonists appear to provide metabolic and hemodynamic protection in KTRs,^80–82^ and their specific roles are discussed below.

#### Infectious GN

Viral and bacterial infections can precipitate glomerular injury in native kidneys^83–86^ and allografts through mechanisms that affect podocyte integrity.^87,88^ In KTRs with chronic hepatitis C virus or hepatitis B virus infection, immune-complex glomerulopathy and complement activation drive secondary podocyte stress and proteinuria, while HIV and, more rarely, BK polyomavirus can directly infect glomerular epithelial cells, including podocytes, and can also serve as targets of infection in advanced disease.^88–90^ In kidney allografts, cytomegalovirus infection can induce endothelial injury with secondary podocyte dysfunction.^90,91^

Severe acute respiratory syndrome coronavirus 2 (SARS-CoV-2) infection represents a novel cause of infection-related glomerular injury, MCD, FSGS, membranous glomerulopathy,^92,93^ and collapsing glomerulopathy, particularly in patients with high-risk apolipoprotein L1 (APOL1) genotype by multiple mechanisms.^90,94–99^ Interestingly, Shetty *et al*.^100^ reported that a transplant recipient carrying a high-risk APOL1 genotype (homozygous variant) developed collapsing glomerulopathy during SARS-CoV-2 infection in an allograft from a donor who carried a low-risk APOL1 genotype (heterozygous variant). This suggests that underlying mechanisms of podocyte injury in the transplant context, and specifically the role of the recipient APOL1 genotype inducing SARS-CoV-2 glomerulopathy need to be fully elucidated.

#### Alloimmune Podocyte Injury

Transplant glomerulopathy (TG) is a distinct allograft lesion, characterized by GBM duplication or multilayering on light microscopy or electron microscopy (in the absence of immune deposits). Clinically, it presents with edema, proteinuria, rising creatinine, and eventually allograft loss. Several pathogenetic factors are at play in TG.^101^ While endothelial damage is likely the initiating event, subsequent injury to podocytes possibly through interruption of endothelial-podocyte crosstalk mechanisms, leading to podocytopenia.^102^ Endothelial injury can result from HLA and non-HLA DSA, and TG is a part of chronic antibody-mediated rejection per the Banff classification.^103^ Nearly 50% of patients with TG had DSA,^104^ most often class 2.^105^ Interestingly, like allotransplantation, pig to rhesus xenotransplantation also reported that long surviving recipients (>300 days)^106^ developed antiswine leukocyte HLA DQ antibodies and TG, suggesting that like allotransplantation, late graft failure was due to TG with positive DSA. In a mouse model of kidney transplant (KTx),^107^ major-histocompatibility-complex mismatched transplants with increased cold ischemia developed class 2 DSAs with macrophage infiltration in glomeruli. By day 60, these allografts had extensive glomerular injury including segmental/global sclerosis, thickened capillary loops, thrombotic microangiopathy, mesangial cell lysis—akin to TG. Pretransplant B-cell depletion reduced C4d deposition and macrophage infiltration confirming an alloantibody mediated process. During antibody-mediated injury, DSAs work synergistically with NK cells,^108^ through antibody-dependent cell-mediated cytotoxicity. Histologically, manifested as MVI and glomerulitis—hallmarks of immune injury in the Banff schema.

In a single-center study, among 1036 biopsies, 5.1% had TG. Within that cohort, 73% had evidence of alloantibody mediated injury in the form of anti-HLA antibody or positive peritubular capillary C4d staining,^109^ implying that at least 30% of TG cases have non-HLA antibodies or alternative mechanisms. Dinavahi *et al*.^110^ used protein microarray to compare antibodies in pretransplant and post-transplant sera from cohorts of patients with and without TG. They found reactivity to peroxisomal-trans-2-enoyl-coA-reductase strongly associated with development of TG. Angiotensin II type 1 receptors are expressed on podocytes,^111^ and anti-angiotensin II type 1 receptor (AT1R) antibodies are associated with rejection.^112^ A potential association between anti-AT1R and post-transplant FSGS was described and nearly half of the patients with positive AT1R antibodies had proteinuria and/or FSGS at the time of anti-AT1R detection.^113^ Similarly, Agrin is a heparan sulfate proteoglycan expressed on GBM. Antibodies to agrin were found in patients with TG (these may be autoantibodies).^114^ Major histocompatibility complex class 1–related chain A (MICA) antigens are surface glycoproteins present on endothelial and epithelial cells among others. In a study of KTRs, anti-MICA antibodies have been linked to higher rejection and reduced allograft survival compared with those who were negative for the antibody.^115^ In a study that evaluated the immunologic effect of A5.1 mutation of MICA *008 allele, there was an association between donor MICA A5.1 and both anti-MICA sensitization and increased proteinuria in recipients.^116^ Although no causal mechanistic data exist for the association of non-HLA antibodies with podocyte injury and TG, these clinical associations raise plausibility of a causal association in some cases of TG.

Recent data show that MVI arises from both antibody-dependent and antibody-independent nonself response mechanisms. Notably, MVI occurs through recipient NK cell-mediated responses of missing-self in the complete absence of demonstrable antibodies,^117^ and MVI may be refractory to calcineurin-based immunosuppression.^118^Large clinical data demonstrated that MVI significantly increases the risk of progression to TG,^119^ underscoring the role of nonself responses in accruing podocyte injury.

### Mechanism of Podocyte Injury in Grafts, Early Hypertrophic Stress as a Cause of Later Graft Failure

Model systems demonstrate that sustained podocyte hypertrophic stress leads to podocytopenia and FSGS.^120^ Yang, Wiggins, and colleagues demonstrated that KTx correlates with podocyte hypertrophy and detachment,^121^ likely due to the transition from two kidney state to one kidney state. They measured podocyte density on preimplantation kidney biopsy (donor two-kidney state), and protocol biopsies in the recipient at 3 and 12 months post-transplantation (adaptive one-kidney state). They found that podocyte nuclear density was significantly reduced, while glomerular volume, and therefore glomerular volume served by each podocyte, increased post-transplant. As a surrogate of podocyte detachment, urine podocin:to creatinine ratio (UPodCr ratio) was measured in this one kidney and two kidney states (*n*=737 samples). The UPodCr ratio was six-fold higher in kidney recipients (*P* < 0.001) and persisted >5 years post-transplant (making it unlikely that native kidneys were the source of urine podocin). In a subgroup analysis, KTRs with eGFR >60 ml/min had near normal UPodCr ratio. By contrast, patients with TG and glomerular diseases had significantly higher UPodCr ratios.^121^ To test whether GS drives late allograft loss, Naik and colleagues analyzed urine mRNA markers from 260 KTRs ranging (immediate post-transplant to 19 years post-transplant, with 87 healthy living donors serving as the control). They found that the urine Aquaporin2: creatinine ratio increased initially but stabilized over time to a level similar to controls. UPodCr ratio increased rapidly initially (presumed due to hypertrophic stress), trended toward control, but then increased steadily suggesting later onset glomerular injury. Urine nephrin: creatinine ratio remained below normal level after transplant, while the UPodCr ratio increased suggesting podocyte loss. Another study by the same group also explored the hypothesis that donor-recipient size mismatch, that is, larger recipient size with a smaller donor kidney size, would be associated with greater podocyte loss because of hypertrophic stress. Here, the UPodCr ratio (representing podocyte stress and loss) was positively associated with recipient body mass index and negatively associated with donor body mass index validating the hypothesis that donor-recipient size mismatch at transplant induce early podocyte injury^122^ and loss. Naik and colleagues assessed podocyte stress and systemic BP changes in a well-characterized cohort of healthy kidney donors. Higher mean arterial pressure (MAP) even within normal ranges (70–110 mm Hg) was associated with higher UPodCr ratio. Interestingly, a significant relationship was found between MAP and podocin:aquaporin ratio (*P*= < 0.0001).^123^ Importantly, the observed injury pattern was preferentially glomerular, as indicated by elevations in intrarenal nephrin and podocin transcripts, but not in tubular injury markers such as kidney injury molecule-1.^123^ These findings suggest that even mild increases in MAP within the normal range impose hemodynamic stress on podocytes.

These results indicate that maladaptive hypertrophic responses in the single-kidney state impair long-term graft outcomes, underscoring the need to identify their drivers to develop targeted therapies.

Based on prior data showing insulin-like growth factor-1 (IGF-1) drives early growth response to uninephrectomy,^124,125^ our group evaluated whether IGF-1 signaling mediates early hypertrophic responses.^126^ Using population, clinical, genetic data, and single-cell RNA-Seq analysis, we found a strong temporal relationship between population level imputed IGF-1 level at the time of transplant and allograft longevity from the United Network for Organ Sharing registry. In a clinical cohort of 216 patients, measured IGF-1 exposure was associated with death-censored graft failure—especially relevant in settings of smaller kidney dose. Analysis of two well-phenotyped transplant cohorts showed that possessing even one risk allele of an IGF1-sSNP, rs35767, was associated with 50% increased risk of death-censored graft failure (HR 1.50, *P* = 0.01). Rs35767 in IGF-1 is known to have regulatory effects on IGF-1 expression,^126^ together connecting IGF-1 levels in recipients to graft outcomes. In prior mechanistic data in a rat model of uninephrectomy, inhibiting angiotensin-converting enzyme or IGF-1 reduced glomerulomegaly if deployed before or during nephrectomy, rather than when used after uninephrectomy, pointing to the critical role of time of compensatory kidney and glomerular growth in determining outcomes.^127^ Hence, these data showing a role for IGF1 signaling in early post-transplant hypertrophic changes have potential to affect long-term outcomes in KTRs and in living donors.

In kidney transplantation, ischemia-reperfusion injury (IRI) is an inevitable contributor to graft dysfunction.^128^ IRI enhances immunogenicity, predisposing to T-cell–mediated and antibody-mediated rejection^129,130^ and drives long-term interstitial fibrosis and tubular atrophy.^131^ Experimental evidence highlights mitochondrial injury and altered podocyte energy metabolism as key drivers of proteinuria and fibrosis, while hypoxic and oxidative stress compromise podocyte adhesion and cytoskeletal integrity.^128,132,133^ The extent to which IRI accelerates podocyte loss during the early post-transplant period—when hypertrophic stress is also present—remains unclear. Human studies quantifying IRI's role in early post-transplant podocyte loss are limited; however, murine models enable precise control of each insult, and separation of ischemic, hypertrophic, and allo-injury early post-transplant.

### Modulation of Post-Transplant Podocyte Injury by Pharmacologic Agents

Immunosuppression remains the cornerstone for preventing and treating graft rejection in kidney transplantation and for treating glomerular and podocyte diseases.^134^ However, these therapies are associated with direct podocyte effects, some of which can be detrimental.^11^

#### CNIs

CNIs are first-line immunosuppressants in kidney transplantation and are often used in glomerular diseases.^135^ Their canonical action is the suppression of *T*-cell activation through the inhibition of calcineurin-mediated nuclear factor of activated *T* cells dephosphorylation.^136,137^ Calcineurin is expressed in podocytes, where it regulates slit diaphragm proteins including nephrin and podocin.^138–141^ Under physiologic conditions, calcineurin prevents synaptopodin dephosphorylation and cathepsin L–mediated degradation, preserving actin organization, limiting foot-process effacement, and reducing podocyte apoptosis.^139^ These cytoskeletal effects contribute to CNIs' antiproteinuric action.^142^ However, despite podocyte protective effects, prolonged exposure causes arteriolar hyalinosis, tubulointerstitial fibrosis, and progressive allograft dysfunction, contributing to CKD and ESKD.^143–145^

#### mTORis

As discussed above, podocyte mammalian target of rapamycin (mTOR) signaling regulates hypertrophy and cytoskeleton and organization.^33,146^ Podocyte mTOR activation has been observed with diabetic nephropathy,^147^ and excess podocyte-specific mTOR activation alone induced FSGS.^33^ Controlled inhibition of mTORC1 can mitigate maladaptive hypertrophy and restore autophagic flux under stress conditions.^148^ Dual inhibition of mTORC1 and mTORC2 may provide additional cytoprotection.^149^ Clinically, mTORis are a second-line agent used to reduce CNI exposure and lower tumor incidence.^150^ Despite these potential benefits, excessive mTOR inhibition can be harmful. Chronic mTORC1 blockade may impair lysosomal function, disrupt autophagy, and promote insulin resistance with altered glucose homeostasis.^151^ mTORis are associated with proteinuria, particularly in transplant patients with reduced nephron number or low baseline eGFR.^152^ They also downregulate slit diaphragm proteins such as synaptopodin, podocin, and nephrin, leading to podocyte apoptosis, especially when patients are switched from CNI-based regimens.^153,154^ Thus, while partial mTOR inhibition can reduce podocyte hypertrophic responses and preserve podocyte integrity, overinhibition carries a risk of accelerating podocyte injury, loss, and FSGS.

#### Abatacept and Belatacept

Targeting the CD80 (B7-1) costimulatory pathway offers a novel approach for podocyte protection. Normally absent on podocytes, CD80 is upregulated in diabetic nephropathy, immune injury, toxin exposure, and relapsing MCD.^154,155^ Abatacept, a CTLA-4-Ig fusion protein, binds CD80 on podocytes and restores *β*1-integrin–mediated adhesion, thereby preventing cytoskeletal disorganization and maintaining slit diaphragm integrity.^156^ In B7-1–positive patients with recurrent or primary FSGS, abatacept has been associated with partial or complete remission of proteinuria, whereas patients lacking B7-1 expression showed no response, underscoring the importance of biomarker-guided therapy.^156,157^

Belatacept, a high-avidity CTLA-4-Ig fusion protein, is widely used in transplantation.^158^ Data on proteinuria after CNI conversion are mixed: some studies reported higher risk of proteinuria despite improved allograft survival,^159–161^ while others showed stability. In a phase Randomized Controlled Trial of KTRs with <1 g/g proteinuria, urine protein creatinine ratio remained stable at 24 months.^162^ Similarly, in a European cohort of 219 recipients converted from CNI or mTORi, the mean proteinuria was unchanged at 12 months after conversion.^163^ In a pilot trial of 15 proteinuric KTRs, conversion reduced proteinuria by ≥25% in more than half of patients, with stable eGFR and graft survival up to 5 years.^164^ Additional studies also demonstrated improved eGFR compared with CNI-treated controls, although rejection occurred in approximately 13% of cases and fibrosis progression was observed.^165^ These findings must also be interpreted in context of the podocyte protective effects of CNIs, which are lost when converted to Belatacept. Overall, costimulatory blockade, whether with belatacept in transplantation or abatacept in proteinuric glomerular disease can stabilize kidney function without significantly worsening proteinuria and may improve long-term graft survival.

#### Renin–Angiotensin–Aldosterone System Blockade

In CKD, renin–angiotensin–aldosterone system blockade reduces proteinuria and preserves podocyte integrity.^166^

Observational studies and systematic reviews, suggested that angiotensin-converting enzyme inhibitors and angiotensin receptor blockers confer benefits in KTRs, with reductions in BP, proteinuria, cardiovascular risk,^167^ and improved graft survival.^168^ In one large retrospective cohort of 2031 KTRs, angiotensin-converting enzyme inhibitors/angiotensin receptor blockers therapy was associated with a 10-year graft survival of 59% compared with 41% without treatment.^168^ Randomized controlled trial data, however, remains limited. In the ramipril trial,^169^ 213 proteinuric KTRs were randomized to ramipril or placebo for a mean of 4 years; no significant difference was observed in the composite end point of doubling of serum creatinine, ESKD, or death, although ramipril modestly reduced proteinuria and BP and had higher discontinuation rates due to anemia and hyperkalemia. The Study on Evaluation of Candesartan Cilexetil after Renal Transplantation (SECRET) trial of candesartan^167^ enrolled 502 recipients and was stopped early due to low event rates but showed improved BP control and reduced proteinuria, while graft survival benefits did not reach significance.

While renin–angiotensin–aldosterone system blockade clearly benefits proteinuric CKD and cardiovascular disease, its graft-protective effect in KTRs remains uncertain—likely reflecting short follow-up, underuse, adverse effects, or fundamental differences in podocyte injury between native and transplanted kidneys. Current guidance supports use in KTRs with cardiac indications or albuminuria^168–172^

#### SGLT2 Inhibitors

SGLT2 inhibitors confer podocyte and kidney protection by enhancing natriuresis, restoring tubuloglomerular feedback, and lowering intraglomerular pressure.^173–179^ Large trials and meta-analyses show that SGLT2 inhibitors slow CKD progression and reduce cardiovascular risk, in both diabetic and nondiabetic populations.^180–183^ A recognized concern is increased bacterial and fungal genitourinary infections,^184^ which is relevant in immunosuppressed patients. In KTRs, short-term use appears safe and well tolerated, with improvements in metabolic parameters, stable tacrolimus levels, and no major increase in adverse events.^185^ In a review of nine KTR studies, SGLT2 inhibitor use was associated with improved hemoglobin A_1c_, preserved eGFR, and no major immunosuppressive drug interactions; urinary tract infections occurred in approximately 13%–15%.^185^ Several trials are ongoing, including SGLT2 Inhibitor Safety and Efficacy on Kidney Allograft Function in Non-Diabetic Kidney Transplant Recipients (SGL-TX-GFR; nondiabetic KTRs, Denmark), Empagliflozin Treatment in Kidney Transplant Recipients (SEKTR; empagliflozin in diabetic and nondiabetic KTRs, VA), EffIcacy, mechaNisms and saFety of SGLT2 INhibitors in kIdney Transplant recIpients (INFINITI2019; dapagliflozin versus placebo with BP as primary outcome), and The Efficacy, Mechanism & Safety of SGLT2 Inhibitor & GLP-1 Receptor Agonist Combination Therapy in Kidney Transplant Recipients (HALLMARK; Toronto, SGLT2i GLP-1 combination in KTRs; ClinicalTrials.gov). Long-term safety in KTRs needs to be studied. Specifically, use in TG and transplant-specific proteinuric syndromes needs to be better defined.

#### GLPs

In diabetic nephropathy models, liraglutide directly protected podocyte by stabilizing the cytoskeleton and attenuating NOD-like receptor protein 3 inflammasome-driven pyroptosis, outperforming insulin alone.^186^ The Research Study to See How Semaglutide Works Compared to Placebo in People With Type 2 Diabetes and CKD (FLOW) trial (*n*=3533) showed that semaglutide in diabetics with CKD reduced major kidney events by 24% (HR, 0.76), slowed eGFR decline (approximately 1.16 ml/min per 1.73 m^2^ per year), reduced albuminuria, and lowered cardiovascular and all-cause mortality, despite more gastrointestinal-related discontinuations.^187^ GLP-1 receptor agonists have recently shown promise in KTRs with preexisting diabetes. In a large US cohort, post-transplant GLP-1 drug use was associated with significantly lower risks of graft loss and mortality, albeit with an observed increased risk of diabetic retinopathy. These findings suggest that beyond metabolic control, GLP-1 receptor agonists may confer kidney-protective effects relevant to podocyte health, warranting further mechanistic studies and prospective trials in the transplant setting.^188^

#### Glucocorticoids

Glucocorticoids directly influence podocytes through activation of the glucocorticoid receptor and nuclear translocation, modulating transcription beyond immune suppression.^154,189,190^ Their protective effects are mediated by stabilization of the actin cytoskeleton, achieved through enhanced polymerization, stress fiber reinforcement, and induction of stabilizing proteins as Hsp27, *α*B-crystallin, and ciliary neurotrophic factor, which together increase resistance to cytoskeletal toxins through RhoA signaling.^189,191–193^ They also lower proinflammatory cytokine levels (IL-6, IL-8) and indirectly mitigate podocyte injury.^194–196^ While high-dose steroids are the first-line treatment in primary FSGS, their use remains limited in recurrent FSGS where plasma exchange is first-line.^197^

## Summary

Podocytes remain vulnerable to immune, mechanical, and metabolic stressors present in the transplant setting. These insults contribute to progressive podocyte loss, accelerating injury and compromising allograft survival. Because podocyte function can be pharmacologically modulated, they represent a promising therapeutic target. Overall, podocyte injury emerges as a common pathway in chronic allograft failure, and elucidating its mechanisms will be key to improving long-term outcomes.
